# A cross-sectional study assessing the self-reported weight loss strategies used by adult Australian general practice patients

**DOI:** 10.1186/1471-2296-13-48

**Published:** 2012-05-30

**Authors:** Sze Lin Yoong, Mariko Leanne Carey, Robert William Sanson-Fisher, Catherine D’Este

**Affiliations:** 1 Priority Research Center for Health Behavior, University of Newcastle, Callaghan, 2308, Australia; 2 Center for Clinical Epidemiology and Biostatistics, University of Newcastle, Callaghan, 2308, Australia; 3 Hunter Medical Research Institute, Newcastle, Australia

**Keywords:** Primary health care, Obesity, Weight loss, General practice, Australia

## Abstract

**Background:**

Obesity is a significant public health concern. General practitioners (GPs) see a large percentage of the population and are well placed to provide weight management advice. There has been little examination of the types of weight loss strategies used in Australian general practice patients. This cross-sectional study aimed to describe the proportion of normal weight, overweight and obese general practice patients who report trying to lose weight in the past 12 months, the types of weight loss strategies and diets used as well as the proportion consulting their GP prior to trying to lose weight.

**Methods:**

Adult patients completed a touchscreen computer survey while waiting for their appointment. Responses from 1335 patients in twelve Australian practices are reported.

**Results:**

A larger proportion of obese patients had tried to lose weight in the past 12 months (73%) compared to those who were overweight (55%) and normal weight (33%). The most commonly used strategy used was changing diet and increasing exercise in all BMI categories. Less than 10% used strategies such as prescription medication, over the counter supplements and consulted a weight loss specialist. Low calorie and low fat diets were the most frequently reported diets used to lose weight in those who were normal weight, overweight and obese. Overall, the proportion seeking GP advice was low, with 12% of normal weight, 15% of overweight and 43% of obese patients consulting their GP prior to trying to lose weight.

**Conclusions:**

A large proportion of overweight or obese patients have tried to lose weight and utilized strategies such as changing diet and increasing exercise. Most attempts however were unassisted, with low rates of consultation with GPs and weight loss specialists. Ways to assist overweight and obese general practice patients with their weight loss attempts need to be identified.

## Background

Obesity is a significant public health concern, affecting a large proportion of people worldwide. Recent studies have reported that approximately 62% of Australians [1] and 68% of Americans [2] have a body mass index (BMI) of ≥ 25 kg/m^2^. This condition imposes significant burden on both the individual and society and is estimated to cost 21 billion dollars in Australia [3] and up to 147 billion in the United States annually [4]. It is well known that weight reductions of five to 10% of baseline body weight in those who are obese can reduce the risk of diabetes and improve clustering of cardiovascular disease related risk factors [5]. Previous population studies in the United States have reported that a substantial proportion of the population have attempted to lose weight by restricting energy and fat intake [6,7]. In Australia, watching the type of food eaten, reducing dietary fat intake and increasing exercise were the most common weight loss strategies reported in population studies [8].

General practitioners (GPs) see more than 80% of the population at least once a year [9] and are well placed to provide weight management advice. Overweight and obesity are also common among general practice patients, with prevalence rates of as high as 70% being reported in this setting [10]. Given that a large proportion of the population see their GP at least once a year, and that many of these will be overweight or obese, this suggests that there may be potential for GPs to opportunistically provide weight management advice and assistance.

A previous study documented that up to 36% of general practice patients have attempted to lose weight at any time in the past 12 months, with a substantial proportion of normal weight patients attempting weight loss [11,12]. Charles et al reported that diet and/or exercise were the most common strategies used by general practice patients [13]. However, previous studies have provided little detail regarding the specific types of weight loss diets used by patients, and whether or not patients consult their GP prior to commencing a new diet or weight loss strategy. This information is likely to be important to informing the development of interventions and tailoring of weight loss discussions in the primary care setting.

The study conducted by Charles et al relied on practitioners to ask patients regarding previous weight loss attempts and methods used [13]. In contrast, the touchscreen computer health questionnaire used in this study is a novel method of data collection and may provide participants with more privacy when reporting sensitive information. The use of electronic data collection methods has previously been shown to be acceptable in a variety of settings such as oncology wards [14] and primary care [15]. It has been used to collect a range of patient-reported information including quality of life [16], psychosocial distress [14], receipt of preventive care [15] and level of pain [17].

Findings from this study will provide insight into the weight management strategies of general practice patients and may help better inform weight loss discussions that occur in this setting.

This study aimed to describe within the normal weight, overweight and obese category; the proportion attempting to lose weight in the previous 12 months, the types of strategies and diets used as well as the proportion that seek GP advice prior to trying to lose weight.

## Methods

### Setting

This cross sectional study was conducted in twelve general practices in Australia between 11^th^ November 2010 and 11^th^ November 2011.

### Participants

Eligible participants were aged 18 years and above, able to read and understand English sufficiently to complete the survey; and physically and mentally able to provide consent.

### Study procedures

A research assistant was present in the surgery and approached consecutive adult patients presenting for care. The research assistant administered one of three surveys on a touchscreen computer to consenting patients. Every third patient received the same survey. Results from the weight loss survey are presented in this paper. If a patient presented for care while the touchscreen computers were in use, the research assistant did not approach that patient. Participants were able to exit the survey if they were called in for their appointment. The research assistant recorded the gender of all invited patients. *Equipment:* A commercial program, Digivey survey suite software (CREOSO - Digivey Survey Center, Phoenix, Arizona) was used to program the survey. The survey was administered using DELL Latitude XT2 touchscreen computers.

The survey was designed to have a Flesh-Kincaid score of 8 in order to minimize the number of patients excluded due to having insufficient English to understand the survey. The survey assessed the following:

### Weight and height

Participants were asked to provide their self reported weight in kilograms (kgs) or stones and height in feet/inches or centimetres (cm). Participants were categorized as underweight (BMI <18.5 kg/m^2^), normal weight (BMI ≥18.5 kg/m^2^ and <25 kg/m^2^), overweight (BMI ≥ 25 kg/m^2^ to BMI <30 kg/m^2^) or obese (BMI ≥ 30 kg/m^2^) [18].

### Demographics

Participants provided information on their age, gender, ethnicity and highest level of education completed.

### Weight change attempts

Participants were asked whether they had tried to change their weight in the last 12 months.

### Types of strategies used

Those who had tried to lose weight in the past 12 months were asked about the specific types of strategies utilized. Participants were asked to select all strategies that applied to them from the following options: (Professional weight loss center programs, Prescription medications, Over-the-counter supplements, Increased exercise, Changed diet, Consulted a weight loss specialist, and Other).

Professional weight loss center programs refer to commercial weight loss programs where lifestyle change is often supervised by weight loss consultants. Prescription medication refers to all medication prescribed by a medical doctor including Dietyhlpropion, Phentermine, Sibutramine and Orlistat. Over-the-counter supplements include all non-prescribed herbal/non-herbal weight loss supplements (e.g. guarana and weight loss pills). Increased exercise refers to any intentional attempts to increase levels of activity to change weight and changing diet refers to intentional dietary changes to produce weight loss. Consulting a weight loss specialist refers to consulting dietary or physical activity specialists including dietitians or exercise physiologists.

If patients indicated that they had tried to change their diet, information about type of diets tried in the past 12 months was elicited. Response options included specialized meal replacements (including milkshakes, power bars), low calorie diet (reduced overall food intake), Atkins diet (low carbohydrate/high protein diet), low fat diet (reduced fat), detox diet, high fibre diet, celebrity/fad diets or other diets.

### Consultation with GP

Participants who indicated trying to change their weight in the last 12 months were asked if they had consulted their GP prior to attempting to lose weight.

### Ethical approval

This study was approved by the University of Newcastle Human Research Ethics Committee (HREC) (H-2009-0341) and ratified by The University of New South Wales HREC (HREC09393/UN H-2009-0341) and Monash University HREC (CF09/3630 – 200900186).

### Statistical analyses

The proportion of males consenting were compared with non-consenters using Pearson’s Chi squared test. Those in the underweight group were excluded due to the small proportion in this group. Descriptive statistics including frequencies, proportions and 95% confidence intervals (CIs) were calculated for previous attempts to change weight, types of strategies and diet used and those consulting their GP prior to trying to lose weight within the normal weight, overweight and obese categories. All analyses was adjusted for clustering of individuals within practices using svy commands. All analyses were performed using STATA 11.0.

## Results

Overall, 1900 participants were approached to complete the weight loss module and 1620 consented to participate (a consent rate of 85%). 1372 (85%) participants provided data regarding weight and height. Of those who were excluded, 11% (n = 27) did not provide valid weight and height information and 89% (n = 221) were called in for their general practice appointment prior to completion of the weight loss module. An additional 37 participants were excluded as they were in the underweight category. A total of 1335 participants were included in the final analyses. There were no significant differences in the proportion of males consenters and non-consenters (χ^2^ = 2.3251, df = 1, p = 0.765).

The demographic characteristics of included patients are shown in Table 1. There were differences in the proportion of females (F(1.57, 17.26) =12.4380 ; p < 0.001) and age (F(4.25, 46.76) = 5.0127; p = 0.0016) between the normal weight, overweight and obese group.

**Table 1:** Demographic characteristic of study participants by BMI category

**Characteristic**	**Normal** **weight** **(*n*** **= 558)** ***n*(%)**	**Over** **weight** **(*n*** **= 473)** ***n*(%)**	**Obese** **(*n*** **= 304)** ***n*(%)**
Age (yrs)*			
18 – 24	47 (8.4)	19 (4.0)	11 (3.6)
25 – 44	165 (29)	11 (25)	72 (24)
45 – 64	173 (31)	176 (37)	126 (41)
≥ 65	173 (31)	159 (34)	95 (31)
Gender (% female) *	387 (70)	237 (50)	189 (62)
**Ethnicity**			
Caucasian	552 (99)	472 (100)	301 (99)
**Has private health insurance**	312 (61)	262 (61)	155 (56)
**Level of Education (n = 1231)**^**#**^			
Completed HSC and below	212(41)	196 (45)	135 (48)
TAFE or Diploma/University	253 (49)	200 (46)	119 (43)
Postgraduate	42 (8.1)	27 (6.2)	12 (4.3)
Other	11 (2.1)	11 (2.5)	14 (5.0)

*p < 0.05; ^*#*^ Number less than total due to incomplete surveys.

HSC = High School Certificate; TAFE = Technical And Further Education

More than half the participants (58%) [95% CI 49–69] were overweight or obese. Overall, 50% [95% CI 45–56] indicated that they had tried to lose weight, 3.1% [95% CI 2.2-4.4] had tried to put on weight and 47% [95% CI 41–53] had not tried to change their weight in the past 12 months. The proportion of patients who had attempted to lose weight increased between normal, overweight and obese groups (see Table 2).

**Table 2:** Proportion trying to change their weight in last 12 months by BMI category

	**Normal (*n* = 555)**^**#**^ **n(%)[95% CI]**	**BMI Category** **Over weight (*n* = 471)**^**#**^ **n(%)[95% CI]**	**Obese(*n* = 303)**^**#**^ **n(%)[95% CI]**	**Total**
Tried to lose weight	187 (33) [27–41]	260 (55) [48.4 - 60.9]	220 (73) [65–79.0]	667
Tried to gain weight	33 (6.0) [3.7 - 9.4]	6 (1.3) [0.4 - 3.9]	2 (0.7 )[0.4 - 3.3]	41
Have not tried to change weight	335 (60) [54–69]	205 (44) [38–49]	81 (27) [21–34]	621

# Number less than total due to incomplete surveys.

Of the 667 people who had tried to lose weight, 50 (7.5%) [95% CI 5.3 - 11] reported using professional weight loss center programs, 11 (1.7%) [95% CI 0.5 - 4.8] used prescription medication, 41 (6.5%) [95% CI 4.3 - 8.8] used over the counter supplements, 477 (72.3%) [95% CI 67–76] changed their diet, 359 (54%) [95% CI 48–59] increased exercise and 43 (6.5%) [95% CI 4.2 -9.8] consulted a specialist. A small proportion (n = 25 (3.9%) [95% CI 2.6-5.5]) indicated using ‘other’ strategies to lose weight.

The most commonly used strategy in all BMI categories were changing diet and increasing exercise (see Table 3). Less than 10% of participants used prescription medicine, over the counter medicine and consulted a specialist.

**Table 3:** Proportion of participants in each BMI category who utilized each type of weight loss strategy

**Strategies used**	**Normal weight (*n* = 555)**^#*^	**BMI n(%)[95% CI]** **Overweight (*n* = 471)**^#*^	**Obese(*n* = 303)**^#*^
Professional weight loss center programs	6 (3.2) [1.3 - 7.5]	22 (8.5) [5.1- 14]	22 (10) [5.5 - 18]
Prescription medication	2 (1.1) [0.1 - 9.3]	1 (0.4) [0.0 - 3.6]	8 (3.6) [1.5 - 8.5]
Over the counter supplements	8 (4.3) [2.1 - 8.5]	17 (6.5)[2.9 - 14]	16 (7.3) [4.4 - 12]
Changed diet	140 (75) [66–83]	181 (70) [63–76]	156 (71) [63–77]
Increased exercise	114 (61) [49–72]	137 (53) [43–62]	108 (49) [41–57]
Consulted a specialist	2 (1.1) [0.3 - 4.2]	15 (5.8) [3.6 - 9.3]	26 (6.5) [4.2 - 9.8]

# Number less than total due to incomplete surveys.

^*^Total of column is larger than the number of participants in each group as participants were able to select more than one response.

The most commonly used diets were low calorie diet and low fat diets (see Table 4). The proportion using specialized meal replacements in the obese group was almost double those in the overweight and normal weight group.

**Table 4:** Types of diets utilized by overweight/obese general practice patients compared to non-overweight patients

**Diets**	**Normal weight (n = 140)**^#*^ n(%) [95%CI]	**BMI Category** **Overweight (n = 181)**^#*^ n(%) [95%CI]	**Obese (n = 156)**^#*^ n(%) [95%CI]
Specialised meal replacements	9 (6.4) [3.6 - 11]	14 (7.7) [73.9 - 15]	23 (15) [9.3 - 23]
Low calorie diet	58 (41) [30–54]	71 (39) [31–46]	65 (42) [35–49]
Low carbohydrate/High protein	8 (5.7) [2.9 - 11]	19 (11) [7.9 - 14]	9 (5.8) [3.5 - 9.3]
Detox diet	5(3.6) [0.6 - 19]	9 (5.0) [2.8 - 8.6]	5 (3.2) [0.8 - 12]
High fibre diet	5 (3.6) [1.3 - 9.5]	15(8.3) [5.0 - 13]	15 (9.6) [5.6 - 16]
Low fat diet	41(29) [20–41]	56 (31) [21–43]	64 (41) [30–53]
Fad /celebrity diet	3 (2.1) [0.7 - 6.4]	1 (0.6) [0.0 - 5.0]	1 (0.6)[0.0 - 5.9]
Other	29 (21) [15–28]	35 (19)[14–29]	39 (25)[20–24]

*significant p < 0.05.

^*#*^ Total of column larger than number of participants in each group as participants were able to select more than one response.

Of those who had tried to lose weight in the past 12 months, 138 (21%) [95% CI 17–26] consulted their GP prior to trying any strategies to change their weight. Of those who were obese and had tried to lose weight, 85 (42% [95% CI 36–48]) had consulted their GP prior to trying to change their weight. A smaller percentage of those overweight (n = 37, (15%) [95% CI 10.4 - 21.2]) and normal weight (n = 16 (7.8%)[ 95% CI 3.8 -16]) had consulted their GP.

## Discussion

Overall, 50% of patients indicated trying to lose weight in the past 12 months. This figure is higher than that identified in previous studies arising from the Bettering the Evaluation and Care of Health (BEACH) dataset, which reported that 35% to 37% [12,13] of patients have attempted to lose weight in the previous 12 months. As part of data collection in the BEACH program, GPs ask their patients about weight loss attempts and types of strategies used. It is possible that patients may have been reluctant to discuss weight loss attempts with their GPs, thus resulting in a lower proportion reporting trying to lose weight. In contrast, the use of a touchscreen computer survey may be a less confronting method of collecting information. The large proportion attempting to lose weight in the present study may also be a result of the increasing prevalence of overweight and obesity in the population [1], although other studies have not noted increases in weight loss attempts despite escalating rates of obesity [6]. Alternatively, social media campaigns promoting healthy weight including “Measure Up” and ‘Swap It Don’t Stop it” launched in Australia at the time of data collection may have increased participant awareness regarding healthy weight. With the increased focus on weight reduction, media messages (including entertainment and advertising) may have also affected participants’ attitudes towards weight loss. Additionally, the stigma faced by those who are obese has been well documented and may be a strong motivator for attempting to lose weight in this group [19].

Also worth noting is that one third (33%) of healthy weight patients indicated trying to lose weight in the last 12 months. Attempts to lose weight in this group could be due to dissatisfaction with current weight or having an unrealistic expectation of ideal body weight [20]. There needs to be attempts to promote the maintenance of healthy weight among those not overweight.

Similar to previous studies [6,13], the most common strategies used by participants were changing diet and increasing exercise. Despite being key components to weight loss, almost half of those who were in the overweight or obese group and had attempted to lose weight did not increase their exercise and approximately 30% did not change their diet. This could be attributed to discouragement from previous failed attempts to change lifestyle habits or difficulty in engaging in exercise due to physical conditions such as osteoarthritis and chronic pain which are more prevalent among those with excess weight [20].

Only a small percentage of general practice patients had previously used prescription medicine. While the use of prescription medicine has been shown to be effective in producing moderate weight loss [21], this strategy may be less acceptable to patients and is associated with side effects such as palpitations, tremors and excess sweating [22].Additionally, the removal of sibutramine from the Australian market in late 2010 may have affected the proportion being prescribed medication for weight loss.

Few participants in the overweight and obese group used strategies where weight loss attempts were assisted by either health care providers (including weight loss specialists) or non-health care providers (including weight loss centres). Previous studies have shown that weight loss counselling delivered by dietitians is effective in producing clinically significant weight loss in general practice patients [23]. Those who consult a specialist might be more likely to receive recommendations in line with best evidence guidelines. There is also some evidence to suggest that the use of structured commercial programs is effective in producing weight loss, with a study reporting that referral by primary care provider to Weight Watchers produced significantly more weight loss compared to usual care [24]. A recent randomized controlled trial (RCT) reported that referrals to commercial based programs produced significantly more weight loss when compared to primary care based specialist delivered program [25]. It appears that a large proportion of those in the overweight and obese group have attempted to lose weight without seeking help from weight loss providers that may have been able to assist them with achieving weight loss.

The most commonly used diets were low calorie and low fat diets as well as meal replacements. As overall energy reduction is needed for weight loss, it is reassuring to note that a large proportion of overweight and obese patients in this study reported reducing their overall calorie intake using a low calorie diet. More than 30% of patients used a low fat diet. While limiting dietary fat may result in some weight loss, guidelines recommend overall calorie reduction rather than restriction of individual macronutrients [26]. An overconsumption of low fat products may also lead to increased energy intake as consumers perceive these products to be healthier [27]. Meal replacements were used by 13% of obese participants. Meal replacements are a promising strategy for weight loss as it provides a structured way of reducing overall calories consumed. A study in general practice patients reported that use of meal replacements with dietitian counselling resulted in almost 10 kg weight loss [28]. Only a small proportion of general practice patients (<5%) reported using diets that could be potentially be harmful to their health (i.e. detox and celebrity diets).

Only 21% of patients consulted their GP prior to trying any strategies to change their weight. This low proportion seeking GP advice reflects previous findings that only 15% of overweight or obese general practice patients sought GP advice to lose weight [13]. Despite findings indicating that more than 50% of patients would consult their GP for weight management advice [11], the proportion seeking GP advice is low. This discrepancy between those wanting advice and those seeking care may be due to patient ambivalence about how prevention fits into a typical consult as well as perceptions that the GP’s role is more focused on dealing with the presenting issue [29]. The role of the GP in weight management has yet to be defined with some arguing that over presentation to health care professionals can result in the medicalization of obesity, leading to the use of more clinical methods of management (including pharmacology and surgery) [30]. GPs are however, a valuable and accessible source of information regarding evidence-based weight management strategies and the involvement of GPs in intervention studies have been shown to increase retention and adherence to weight loss strategies [23].

### Strengths and limitations

This study relied on patient self report to obtain information regarding BMI (weight and height), weight loss attempts and strategies. As self reported is associated with social desirability bias, this may have resulted in an overestimation in proportion trying to lose weight and underestimation of using popular diets known to be hazardous to health (e.g. fad/celebrity diets). However, the use of the touchscreen computer to collect information may have provided patients with more privacy, thus reducing misreporting attributed to social desirability bias. Additionally, studies have also shown that patients tend to underreport weight and overreport height, leading to an underestimation of BMI [31]. Measurements conducted in a subsample of participants (unpublished data) identified a low mean error between self-reported and measured weight and height. This study had a large sample size and high patient consent rate (85%), suggesting that it is likely to be representative of adult patients presenting for care in the participating practices. Findings from this study are not generalizable to non-English speaking patients as this group was specifically excluded from the study. However, given that less than 10% of those presenting for care are from non-English speaking backgrounds [9], it is likely that findings reported here are representative of those typically presenting for care to these practices.

### Practice implications

Patients who have received advice from a health care professional to lose weight are more likely to attempt to lose weight [32]. This suggests that with a large proportion of overweight and obese patients attempting to lose weight in the past 12 months, there is an opportunity for GPs to play a greater role in assisting patients with weight loss attempts. To ensure feasibility in the time-pressured general practice setting, it is necessary to establish systems to support GPs in this role. Practice nurses may play an important role in assisting with identification of overweight or obesity as well as scheduling follow up reviews where appropriate [33,34]. Future studies are needed to examine the way in which primary care providers can assist overweight and obese patients with their weight loss attempts in order to produce weight loss.

## Conclusion

A large proportion of overweight and obese patients report having tried to lose weight in the past 12 months. Strategies such as changing diet and increasing exercise were commonly reported. Most attempts however were unassisted, with low rates of consultation with GPs and weight loss providers. Ways to assist overweight and obese patients with their weight loss attempts need to be identified.

## Competing interest

The authors declare that they have no competing interests.

## Authors’ contributions

SY, MC, CD and RSF all participated in conception of the study and survey design. SY conducted some data collection and initial data analysis. SY, MC and CD had input into the statistical analysis. All authors offered comments on the draft of the manuscript and approved the final copy for submission.

## Pre-publication history

The pre-publication history for this paper can be accessed here:

http://www.biomedcentral.com/1471-2296/13/48/prepub
